# The Generative Adversarial Brain

**DOI:** 10.3389/frai.2019.00018

**Published:** 2019-09-18

**Authors:** Samuel J. Gershman

**Affiliations:** Department of Psychology and Center for Brain Science, Harvard University, Cambridge, MA, United States

**Keywords:** bayesian inference, delusions, consciousness, generative adversarial networks, perception

## Abstract

The idea that the brain learns generative models of the world has been widely promulgated. Most approaches have assumed that the brain learns an explicit density model that assigns a probability to each possible state of the world. However, explicit density models are difficult to learn, requiring approximate inference techniques that may find poor solutions. An alternative approach is to learn an implicit density model that can sample from the generative model without evaluating the probabilities of those samples. The implicit model can be trained to fool a discriminator into believing that the samples are real. This is the idea behind generative adversarial algorithms, which have proven adept at learning realistic generative models. This paper develops an adversarial framework for probabilistic computation in the brain. It first considers how generative adversarial algorithms overcome some of the problems that vex prior theories based on explicit density models. It then discusses the psychological and neural evidence for this framework, as well as how the breakdown of the generator and discriminator could lead to delusions observed in some mental disorders.

## 1. Introduction

Our sensory inputs are impoverished, and yet our experience of the world feels richly detailed. For example, our fovea permits us access to a high fidelity region of the visual field only twice the size of our thumbnail held at arm's length. But we don't experience the world as though looking through a tiny aperture. Instead, our brains feed us a “grand illusion” of panoptic vision (Noë et al., 2000; Chater, 2018; Odegaard et al., 2018). Similarly, we receive no visual input in the region of the retina that connects to the optic nerve, yet under normal circumstances we are unaware of this blind spot. Moreover, even when we receive high fidelity visual input, we may still fail to witness dramatic changes in scenes (Simons, 2000), as though our brains have contrived imaginary scenes that displace the true scenes.

There is a standard inferential explanation of these and many other illusions (e.g., Gregory, 1980), which holds that our percepts reflect beliefs about the world rather than raw sensory information. In modern computational models of perception, these beliefs are typically conceptualized as probability distributions over some hypothesis space conditional on the sensory input, as stipulated by Bayes' rule (Knill and Richards, 1996):

(1) $$\begin{array}{l} P\left(z|x\right)=\frac{P\left(x|z\right)P\left(z\right)}{\sum_{z^{\prime }}P\left(x|z^{\prime }\right)P\left(z^{\prime }\right)}, \end{array}$$

where *P*(*x*|*z*) is the likelihood of the data *x* given hypothesis *z*, *P*(*z*) is the prior probability of *z*, and *P*(*z*|*x*) is the posterior probability. While the Bayesian framework has considerable merit, it does not seem to provide adequate answers to several questions.

First, how can we explain the phenomenology of illusion: why do some illusions feel *real*, as though one is actually seeing them, whereas other inferences carry information content without the same perceptual experience. For example, Ramachandran and Hirstein (1997) use the example of gazing at wallpaper in a bathroom, where the wallpaper in your visual periphery is “filled in” (you subjectively experience it as high fidelity even though objectively you perceive it with low fidelity), but the wallpaper behind your head is not filled in. In other words, you *infer* that the wallpaper continues behind your head, and you may even know this with high confidence, but you do not have the experience of *seeing* the wallpaper behind your head. Thus, the vividness or “realness” of perceptual experience is not a simple function of belief strength. So what is it a function of?

Second, how can we explain the peculiar ways that the inferential apparatus breaks down? In particular, how can we understand the origins of delusions, hallucinations, and confabulations that arise in certain mental disorders? While Bayesian models have been developed to explain these phenomena, they fall short in certain ways that we discuss later on.

In this paper, we argue that these issues can be addressed by thinking about Bayesian inference from a different algorithmic perspective. The basic idea is that a “generator” draws samples from the generative model, which are then fed, along with samples of real sensory data, into a “discriminator” that tries to figure out which samples are real and which are fake. These two components are in a kind of arms race: the generator is trying to produce samples that trick the discriminator into incorrectly classifying them as real, and the discriminator is trying to learn how to detect these fakes. If the visual system plays the role of the generator, and our perceptual experience reflects the judgment of the discriminator, then we can begin to understand why the visual system might report things that aren't there, or fail to report things that are there, and why our perceptual experience endorses these false or incomplete reports (see also Lau, 2019). Furthermore, breakdown of the generator and discriminator may explain the origin of false beliefs and percepts in certain mental disorders: a dysfunctional generator can produce abnormal content, and a dysfunctional discriminator can endorse that content as real.

This “generative-adversarial” interplay is motivated by recent advances in machine learning, which have produced algorithms for learning generative models based on the same idea. In the next section, we summarize the idea more formally. What follows is a rampantly speculative discussion of implications for psychology and neuroscience (note that the article is not proposing any novel computational ideas from the perspective of machine learning). Finally, we apply these ideas to understanding delusions observed in some mental disorders.

## 2. Generative Models: Explicit and Implicit

Generative models can be understood as stochastic “recipes” for generating observed data: first draw a latent variable *z* from the prior *P*(*z*), then draw data from the conditional distribution *P*(*x*|*z*). This generative model can then be inverted according to Bayes' rule to recover a posterior belief *P*(*z*|*x*) about the latent variable conditional on the data. There are two basic problems that any probabilistic information processing system (artificial or biological) must face. The *inference problem* is how to compute the posterior efficiently given constraints on computational resources. The *learning problem* is to update the generative model *P*(*x, z*) in order to better match the empirical data distribution. Learning is limited both by the amount of training data and by the difficulty of searching through the space of probability distributions (typically via gradient-based techniques).

Exact Bayesian inference is intractable for most moderately complex generative models. This means that if we are going to consider expressive generative models, we will need to also consider approximate inference. Historically, approximate inference algorithms have fallen into two families (Gershman and Beck, 2017). One family, Monte Carlo algorithms, approximates the posterior via stochastic simulation. Provided enough samples are drawn, Monte Carlo algorithms can, at least in theory, approximate the posterior arbitrarily well. They can account for a wide range of neural (Buesing et al., 2011; Haefner et al., 2016; Orbán et al., 2016), and behavioral (Sanborn and Chater, 2016; Dasgupta et al., 2017) data. Their main limitation is that they can be woefully inefficient for complex distributions, unless one uses more sophisticated variants that pose challenges for neural and psychological plausibility.

The second family, variational algorithms, approximate the posterior with a simpler parameterized form that is easier to optimize. Variational algorithms have figured prominently in neuroscience, where they underpin the free-energy principle (Friston, 2009), and have also been proposed as psychologically plausible process models (Sanborn and Silva, 2013; Dasgupta et al., 2019). These algorithms are often much more efficient compared to Monte Carlo, which is why they are widely used in machine learning. However, because of the simplified parameterization, the optimal approximation will typically be biased (i.e., it won't perfectly capture the true posterior).

A basic limitation of both Monte Carlo and variational algorithms is that they are mainly designed to work with *explicit* generative models: they assume that the likelihood can be evaluated for any data sample. However, there are many complex models that are *implicit* in the sense that they can only be simulated. For example, the drift-diffusion model does not have a tractable closed-form expression for the likelihood function, but samples can be drawn from the generative model. This has motivated various forms of “likelihood-free” algorithms (e.g., Diggle and Gratton, 1984; Csilléry et al., 2010; Hartig et al., 2011; Gutmann and Corander, 2016).

**Figure 1:**
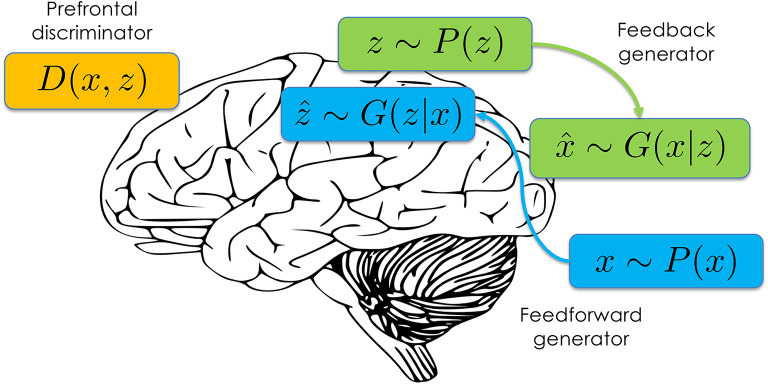
Schematic of the adversarially learned inference architecture mapped onto the brain.

Recently, a new approach to likelihood-free approximate inference has emerged based on a minimax game between a generator *G* and a discriminator *D* (Donahue et al., 2016; Dumoulin et al., 2017).^1^ Both the generator and discriminator are typically implemented as differentiable neural networks. The discriminator takes as input data *x* and latent variable *z*, and outputs the probability that (*x, z*) was drawn from the joint distribution *P*(*x, z*) vs. the generator distribution *G*(*x, z*). The generator consists of two components (Figure 1): a “feedforward” component *G*(*z*|*x*) that samples inferred latent variables ẑ conditional on empirical data *x*~*P*(*x*), and a “feedback” component *G*(*x*|*z*) that samples simulated data $\hat{x}$ conditional on draws from the prior *z*~*P*(*z*). The feedforward component implements the approximate inference engine, efficiently mapping data to samples from the approximate posterior over latent variables. The feedback component implements the learned generative model, mapping latent variables to samples from the observation distribution.

The generator and discriminator are jointly trained to optimize the following “adversarial” objective function:

(2) $$\begin{array}{l} \underset{G}{\min}\;\underset{D}{\max}{\text{E}}_{G\left(z|x\right)P\left(x\right)}\left[\text{log}D\left(x,z\right)\right]+{\text{E}}_{G\left(x|z\right)P\left(z\right)}\left[\text{log}(1-D\left(x,z\right))\right]. \end{array}$$

Intuitively, the generator is trying to fool the discriminator into placing high probability on simulated data and low probability on empirical data, while the discriminator is trying to do the opposite. It can be shown (Dumoulin et al., 2017) that the optimal discriminator for a fixed generator is given by:

(3) $$\begin{array}{l} D^{*}\left(x,z\right)=\frac{G\left(x,z\right)}{G\left(x,z\right)+P\left(x,z\right)}. \end{array}$$

Thus, the discriminator will be at chance when the generator has perfectly approximated the true joint distribution. The optimal generator can also be understood as minimizing the Jensen-Shannon divergence between *G* and *P* (Goodfellow et al., 2014; Dumoulin et al., 2017)^2^.

Adversarially learned inference has two important advantages over standard Monte Carlo and variational approaches. First, as already noted, it can be applied to implicit generative models, which means that these models can be more complex (e.g., parameterized as a deep neural network with an intractable likelihood function). The result is that the quality of the generative model is higher, as measured (for example) in terms of simulated data quality. Second, inference is more efficient than standard Monte Carlo algorithms (it is “amortized” in the form of a learned function that can be quickly evaluated) and can use more flexible approximate posteriors compared to standard variational algorithms^3^.

## 3. Psychological Implications

### 3.1. The Puzzle of Phenomenology

We began this paper with examples from visual perception in which people have the subjective experience of seeing things that are objectively not there (e.g., high acuity in the periphery or in the retinal blind spot). This is sometimes discussed as perceptual “filling-in,” though this term is theoretically tendentious: it suggests something like a neural paintbrush that fills in missing segments on an internal screen, an idea that (Dennett, 1992) has argued is highly implausible. As an alternative, Dennett suggests something more like “paint-by-numbers,” where surfaces are symbolically labeled, and these symbols are interpreted appropriately by downstream computations. Indeed, this is roughly how digital computers typically deal with surfaces.

As a matter of neurophysiology, it turns out that Dennett was incorrect: there really is an interpolation process in low-order visual areas that is retinotopically organized (De Weerd, 2006). The more important point for present purposes is that Dennett's argument doesn't really explain the subjective experience of perceptual filling-in. Either interpolative or symbolic implementations could be compatible with this subjective experience. In essence, the question is why the down-stream interpreter of these representations ascribes “realness” to some representations (wallpaper in front of you, to again use Ramachandran and Hirstein's example) and not others (wallpaper behind you).

Noë et al. (2000) have offered a different line of argument, that we don't actually have the subjective experience of seeing stimuli in the periphery or the blind spot, but rather our phenomenology reflects the knowledge that the relevant stimulus information is available in the environment, and we could (e.g., with eye movements) apprehend that information. This seems somewhat unsatisfactory, because it is basically denying the introspective observation that we experience ourselves as really seeing stimuli in the periphery. It also seems to conflict with psychophysical experiments demonstrating that people are overconfident about how much they see in the periphery (Odegaard et al., 2018). If it was simply a matter of knowing that we *could* see something, not that we actually *do* see something, then there's no reason why we should feel overconfident about our perceptual acuity.

The adversarial framework leads to another way of thinking about these issues. The discriminator is, by design, making ascriptions of “realness” to inputs that are both real and simulated. Meanwhile, the generator is trying its best to feed the discriminator realistic simulations. Thus, if subjective perceptual experience corresponds to perceptual content that has been endorsed as real by the discriminator, then we would have an explanation for why we feel that we see more than we do. Simulations of peripheral visual input are highly compelling. On the other hand, simulations of visual inputs outside the field of vision are not. The generator can trick the discriminator into thinking that it sees wallpaper in front of us, but not behind us.

This perspective has some resonance with higher-order theories of consciousness (Lau and Rosenthal, 2011; Lau, 2019), which hold that conscious awareness is a particular kind of mental state that represents other mental states. The discriminator can be understood as a higher-order representation that represents beliefs (real vs. imagined) about lower-level perceptual representations. On this view, conscious awareness occurs when a decision is made that a perceptual representation is veridical (see also Dehaene et al., 2014).

The adversarial framework contrasts with the interoceptive predictive coding account of Seth et al. (2012), according to which the sense of reality derives from the perception of sensorimotor contingency. While sensorimotor contingency might be one piece of information that the discriminator uses to make its decisions, it can also use other sources of information. For example, people who are unable to move their eyes may experience low sensorimotor contingency, but can still discriminate real from imagined stimuli.

### 3.2. Discriminating Between Reality and Imagination

The adversarial framework posits that a mechanism for discriminating between reality and imagination plays an important computational role in learning and inference. In the psychology literature, the discrimination problem has been studied in the context of *reality testing* (discriminating between real and imagined stimuli in perception) and *reality monitoring* (discriminating between real and imagined stimuli in memory). The most famous example of reality testing is the Perky effect. Perky (1910) presented subjects with dimly illuminated images of objects while subjects were asked to describe the objects, and found that subjects falsely reported these as imagery rather than perception. Segal and Fusella (1970) examined this effect with signal detection techniques, finding that sensitivity was reduced under mental imagery conditions, particularly for perceived and imagined stimuli in the same sensory modality. Many subsequent studies have documented interactions between imagery and perception. For example, Farah and Smith (1983) demonstrated that imagery can facilitate stimulus detection (see also Farah, 1985; Ishai and Sagi, 1995).

The study of reality monitoring has been championed by Johnson and her collaborators (see Johnson and Raye, 1981, for a review of the early literature), who have called attention to the problem that mental images leave traces in memory, and therefore some mechanism must exist to discriminate between these memories and memories of observed stimuli. As we discuss below, this mechanism appears to have a dedicated neural substrate, and dysfunction of this mechanism may underpin cognitive and perceptual symptoms in certain mental disorders. One important set of findings from research on reality monitoring is the identification of factors that people use to discriminate reality from imagination. For example, real stimuli are richer in perceptual and semantic detail, and contain less information about cognitive operations. These are all factors we would expect that a well-designed discriminator could exploit.

## 4. Neural Implications

The architecture shown in Figure 1 lends itself naturally to a systems-level interpretation. The discriminator corresponds to a reality monitoring mechanism that has been frequently attributed to the median anterior prefrontal cortex (see Simons et al., 2017, for a review). For example, this region is activated when subjects are asked to discriminate whether a visual object was previously seen or imagined (Kensinger and Schacter, 2006), and morphological features of this region covary with individual differences in reality monitoring performance (Buda et al., 2011). Moreover, patients with schizophrenia (Garrison et al., 2017) and healthy individuals prone to expression of psychotic and schizotypal traits (Simons et al., 2008) both show reduced activation in this area during reality monitoring.

The “feedback” and “feedforward” terminology was chosen to suggest a mapping onto feedback and feedforward pathways in posterior cortical regions. This is consistent with theories of cortical function that posit a role for feedforward pathways in computing inferences about the latent causes of sensory data, and a role for feedback pathways in computing predictions about upcoming sensory data (e.g., Dayan et al., 1995; Lee and Mumford, 2003; Lochmann and Deneve, 2011). Some theories (e.g., Rao and Ballard, 1999; Friston, 2008) have argued that feedforward pathways convey prediction *errors* rather than predictions. This can be understood as an efficient way to pass predictions up the cortical hierarchy while removing redundant information (see Huang and Rao, 2011).

At the circuit level, an implicit generative model could be implemented as a probabilistic population code (PPC; Ma et al., 2006), which represents a probability distribution via the distribution of spikes across a population. One challenge facing PPCs is that they only support exact inference for relatively simple generative models, such as Kalman filtering and multi-sensory cue combination. Some authors have attempted to generalize PPCs to the approximate inference setting, for example by having the PPCs encode the sufficient statistics of a factorized variational approximation (Beck et al., 2012) or the sufficient statistics of cliques in a graphical model that then pass messages using loopy belief propagation (Raju and Pitkow, 2016). Both of these generalizations limit the kinds of generative models that can be represented. Adversarially learned inference provides potentially another way to work with more flexibly parameterized models. An open problem is to determine what kinds of biologically plausible learning rules could implement optimization of the adversarial objective function.

## 5. Delusions

In the field of cognitive neuropsychiatry, some authors have invoked inferential explanations of delusion formation (Hemsley and Garety, 1986; Corlett et al., 2009; Coltheart et al., 2010; McKay, 2012; Sterzer et al., 2018). According to the “two-factor” version of this idea (see Coltheart et al., 2010), two underlying factors must break down: (i) the input data must be abnormal, and (ii) the hypotheses suggested by the abnormal data must be defectively evaluated. Some patients have an impaired first factor but an intact second factor; these patients have abnormal experiences but do not develop delusions. Coltheart et al. (2010) viewed the evaluation factor as a form of Bayesian inference, but conceded that Bayes' rule is silent about the origin of abnormal data (the first factor). Moreover, the conjectured impairment in the evaluation factor—that patients are unable to assimilate evidence contradicting the delusional belief—runs into trouble. As pointed out by McKay (2012), it doesn't really make sense chronologically why patients would be able to assimilate the abnormal data but not the subsequent contradictory data. As an alternative, McKay suggests that the impairment in the evaluation factor is a bias toward “explanatory adequacy,” whereby the likelihood is overweighted at the expense of the prior. This alternative still leaves the origin of abnormal data unexplained.

In support of the two-factor interpretation, Coltheart et al. (2010) discuss evidence that impairments of abnormal data and abnormal evaluation are dissociable. For example, some patients with damage to the ventromedial prefrontal cortex fail to autonomically discriminate between familiar and unfamiliar faces, as measured by skin conductance, despite their ability to recognize the familiar faces (Tranel et al., 1995). Coltheart et al. view these cases as analogous to Capgras patients, in the sense that both syndromes produce abnormal content, but with the critical difference that Capgras patients develop delusions because of their impaired ability to evaluate the abnormal content, whereas ventromedial prefrontal patients do not develop delusions.

Another example is the Fregoli delusion, which is essentially the opposite of the Capgras delusion: patients perceive strangers as familiar people in disguise. It has been suggested that the underlying mechanism of abnormal content generation is the opposite of the putative mechanism underlying Capgras delusion, namely an over-responsive autonomic response to faces (Ramachandran et al., 1998). Importantly, there are patients who show the same abnormal content generation (strange faces are perceived as highly familiar) but who do not develop delusions (Vuilleumier et al., 2003).

Some theorists have advocated for a “one-factor” predictive coding version of the inferential account (e.g., Corlett et al., 2009; Sterzer et al., 2018), according to which delusion formation arises from a single cause: noisy prediction errors, which register the discrepancy between observations and expectations and drive updating of beliefs. Noise in the prediction errors furnishes the abnormal input data, which in turn drives aberrant belief updating. One potentially problematic aspect of this account is that it seems to require the noise to be quite large in order to produce the kinds of dramatic delusions that have been observed (e.g., believing that family members have been replaced by imposters, as in Capgras syndrome). Although there is evidence for noisy neural signaling in schizophrenia (Winterer and Weinberger, 2004), signal detection analyses of psychophysical performance have indicated that internal noise levels do not differ between schizophrenics and healthy controls (Collicutt and Hemsley, 1981; Bentall and Slade, 1985). Moreover, some disorders (e.g., autism; see Dinstein et al., 2012; Park et al., 2017) have been associated with elevated noise levels but are not reliably associated with delusions (though see van Schalkwyk et al., 2017). Two-factor theorists sometimes posit that the first factor results from a specific neurological impairment (e.g., disconnection between autonomic signaling and face recognition in Capgras syndrome) rather than a general increase in noise, which would be expected to produce a much wider variety of abnormal experiences.

Adversarially learned inference provides a different perspective on these issues. Abnormal content arises from defects in the generator, which cause it to produce simulated data $\hat{x}$ and simulated interpretations ẑ that have low probability under *P*(*x, z*). These simulations are accepted by delusional patients because those patients also have a defect in their discriminator that impairs its ability to tell apart true and simulated samples. Thus, adversarially learned inference can be considered similar to two-factor theory, in the sense that it posits distinct impairments of abnormal content and abnormal evaluation.

The generative adversarial perspective offers a way to correct some of the shortcomings of prior Bayesian accounts. First, it suggests a broad hypothesis about the origin of delusional content (via an abnormal generator), whereas Bayesian models are silent on the origin of delusional content beyond the postulate that prediction errors are noisy. As discussed above, noisy prediction errors seem inadequate to account for both the magnitude and specificity of delusional content. Second, the discriminator directly formalizes ideas about reality monitoring that have been applied to delusions, hallucinations, and confabulations (Bentall et al., 1991; Turner and Coltheart, 2010). In contrast, Bayesian models do not typically postulate any kind of specialized reality monitoring mechanism. While we have focused on delusions, the adversarial account may provide a broader framework that accompanies other kinds of reality distortion like hallucinations. The fact that hallucinations and delusions covary in schizophrenia (Grube et al., 1998) suggests that there may be a common underlying etiology.

## 6. Discussion

This paper has assembled evidence across several disparate domains (perceptual phenomenology, neurobiology, and neuropsychiatry) in favor of a generative adversarial framework for approximate inference. In closing, we consider some broader issues and open questions.

### 6.1. Learning From the Imagination

Adversarially learned inference uses imagination to drive learning, exemplifying a broader class of imagination-based learning models that have been studied in cognitive science. The effects of imagination on learning have been widely documented (see Kappes and Morewedge, 2016, for a review). For example, Tartaglia et al. (2009) demonstrated that perceptual learning can occur through mental imagery, and related results have been observed across many different cognitive and behavioral tasks (Driskell et al., 1994; Gershman et al., 2017). It is unlikely that all imagination-based learning phenomena can be subsumed by the generative adversarial perspective. There are many ways that imagination could be involved in learning that don't involve adversarial interactions between a generator and a discriminator. For example, Niyogi et al. (1998) described how to use image transformations to produce “virtual examples” that can be used as additional training data, and Sutton (1990) developed related ideas for reinforcement learning. Both of these examples are forms of *data augmentation*, a technique widely used in machine learning to improve performance when data are limited (for some recent examples, see Hauberg et al., 2016; Ratner et al., 2017). Interestingly, generative adversarial algorithms have also ben employed for this purpose (Antoniou et al., 2017).

A key assumption of data augmentation algorithms is that the augmented data share certain properties with the true data distribution. In supervised learning, the augmented data must have the same labels as the true data. For example, Niyogi's technique is based on the idea that rigidly defined objects are invariant to rotations and translations. In reinforcement learning, augmented rewards and state transitions can be sampled from a learned model of the environment, as in Sutton's technique. The challenge, then, is to devise a scheme for producing augmented data with the right properties. Adversarially learned inference can be understood as one particular approach to this problem. The generator is not learning directly from the data distribution, but rather from a supervised signal (discriminator inaccuracy) that tells the generator how convincingly it has emulated the data distribution.

### 6.2. Toward a Synthesis of Approximate Inference Algorithms

Another broad issue concerns how we should make sense, and perhaps bring together, the menagerie of ideas about approximate inference in the brain. Adversarially learned inference shares elements of both Monte Carlo and variational algorithms. It uses samples to approximate expectations (as in Monte Carlo algorithms). But it also optimizes an objective function (the Jensen-Shannon divergence) that is closely related to standard variational algorithms (see Nowozin et al., 2016). Some generative adversarial approaches to inference make the connection even more explicit (Huszár, 2017; Mescheder et al., 2017). An interesting direction for future work will be to see whether some more systematic synthesis of these ideas is possible.

### 6.3. Predictions

Generative adversarial approaches to inference make a number of testable predictions. One is that impairment in the discriminator should lead to systematic distortions in learning, since imagined stimuli will be treated as real data. This should lead to generators that produce unrealistic samples, which could be tested by studying statistical learning in patients with prefrontal damage or with schizophrenia.

More broadly, the neural networks that have been developed for artificial intelligence tasks are designed to operate on high-dimensional data like natural images and videos, which opens up the possibility to make predictions about reality monitoring and subjective experience for real-world sensory inputs. For example, one could use them to predict which images are more likely to produce reality monitoring errors or meta-cognitive illusions in the periphery.

## Author Contributions

The author confirms being the sole contributor of this work and has approved it for publication.

### Conflict of Interest Statement

The author declares that the research was conducted in the absence of any commercial or financial relationships that could be construed as a potential conflict of interest.

## Footnotes

^1^The space of generative-adversarial algorithms is much broader than what is covered in this paper. The original formulation (which did not involve inference at all) is due to Goodfellow et al. (2014). The relationship between generative-adversarial inference algorithms and other approximate inference algorithms is discussed in Huszár (2017).

^2^Note that the product rule of probability implies that *G*(*x, z*) = *G*(*z*|*x*)*P*(*x*) = *G*(*x*|*z*)*P*(*z*). However, because the two generator components are parameterized independently, this equality may not hold in practice, except at the optimum of the objective function (provided both components are sufficiently expressive).

^3^Note that amortization can also be applied to variational inference in explicit generative models, so this advantage is not unique.
